# Cumulative culture in nonhumans: overlooked findings from Japanese monkeys?

**DOI:** 10.1007/s10329-017-0642-7

**Published:** 2017-12-27

**Authors:** Daniel P. Schofield, William C. McGrew, Akiko Takahashi, Satoshi Hirata

**Affiliations:** 1 Institute of Cognitive and Evolutionary Anthropology, University of Oxford, Oxford, OX2 6PE UK; 2 School of Psychology and Neuroscience, University of St. Andrews, St. Andrews, KY15 9JH UK; 3 National Museum of Emerging Science and Innovation, 2-3-6 Aomi, Koto-ku, Tokyo, Japan; 4 Wildlife Research Center, Kyoto University, Kyoto, 606-3201 Japan

**Keywords:** Cumulative culture, Japanese macaque, Food processing, Traditions, Ethnography

## Abstract

Cumulative culture, generally known as the increasing complexity or efficiency of cultural behaviors additively transmitted over successive generations, has been emphasized as a hallmark of human evolution. Recently, reviews of candidates for cumulative culture in nonhuman species have claimed that only humans have cumulative culture. Here, we aim to scrutinize this claim, using current criteria for cumulative culture to re-evaluate overlooked qualitative but longitudinal data from a nonhuman primate, the Japanese monkey (*Macaca fuscata*). We review over 60 years of Japanese ethnography of Koshima monkeys, which indicate that food-washing behaviors (e.g., of sweet potato tubers and wheat grains) seem to have increased in complexity and efficiency over time. Our reassessment of the Koshima ethnography is preliminary and nonquantitative, but it raises the possibility that cumulative culture, at least in a simple form, occurs spontaneously and adaptively in other primates and nonhumans in nature.

## Introduction

Nineteenth-century anthropologists such as Tylor (1871) and Morgan (1877) championed ideas of a linear, evolutionary progression of human society through cultural stages from ‘savagery’ to ‘civilization.’ Although this thinking has faded away, some disciplines, such as anthropology, archaeology, and psychology, maintain the view that human society occupies a solitary pinnacle in the animal kingdom (Whiten and van Schaik 2007; Mesoudi 2011, 2016). This thinking occurs in often-cited cases of superlative human achievements: space exploration, modern medical technology, invention of calculus, etc. These cases represent the culmination of the achievements of many persons over multiple generations, each making gradual modifications to the innovative advances that preceded them. The evolutionary process driving this remarkable enhancement is said to be ‘cumulative culture’ (CC), conceived generally as the ever-increasing, additive complexity or efficiency of cultural performance over time. Authors from various academic disciplines assert that CC is what separates *Homo sapiens* from all other living species cognitively and behaviorally (e.g., anthropology, Hill 2009; archaeology, Haidle et al. 2015; psychology, Tennie et al. 2009; Tomasello 2009; primatology, Perry 2009; philosophy, Sterelny 2009; even neuroscience, Somel et al. 2013; but cf. ethology, Whitehead and Rendell 2015).

CC has been characterized as a ‘ratchet,’ yielding progressive innovation and improvement over generations (Tomasello et al. 1993). The process can be seen as repeated inventiveness that leads to incrementally better adaptation; that is, more efficient, secure, convenient, etc. survival and reproduction. The conceptual consensus is that high-fidelity information transmission and cognitively complex social learning in humans facilitates the expression of behavior and the products of behavior, prevents ‘slippage,’ and allows the modification of cultural traits to ratchet up, advancing beyond what any individual could achieve alone (Tennie et al. 2009). Advocates of this position (Galef 2009; Hill 2009; Tomasello 2009) maintain that there is no evidence, either experimental or observational, that any other species possesses CC.

Recent evidence of innovation, transmission, propagation, diffusion, and intergroup variation in behavior in a wide range of species in nature has bolstered arguments for animal culture, stressing continuity between behavioral mechanisms of humans and nonhumans (McGrew 2004; Whitehead and Rendell 2015). Proposed evidence for CC in nonhumans in nature includes tool use in chimpanzees (*Pan troglodytes*; Sanz and Morgan 2009) and New Caledonian crows (*Corvus moneduloides*; Hunt and Gray 2003), social games in white-faced capuchin monkeys (*Cebus capucinus*; Perry 2011), and stone handling (SH) by Japanese macaques (Leca et al. 2007a, 2012). Recent reviews of this evidence have dismissed these claims as flawed and inconclusive, leaving the sceptics unconvinced (e.g., Dean et al. 2014).

All of the above-cited examples (except for SH, see below) rely almost entirely on indirect tests of CC; that is, they are based on inferential, retrospective reconstruction and lack chronological (real-time) evidence of cumulative change. They rely on cross-sectional rather than longitudinal data. For example, termite-fishing tools used by wild chimpanzees at Goualougo are more efficient if they have frayed tips than the unfrayed tips used elsewhere. This suggests that Goulougo chimpanzees have advanced the design of this extractive technology by modifying and so improving their tools’ tips (Sanz and Morgan 2009). But there are not yet enough long-term ethological data, nor a corresponding archaeological record, to test this idea at their study site.

Here, we revisit the well-known early studies of the Japanese monkey (*Macaca fuscata*) at Koshima, as potential candidates for cumulative culture in nonhumans.

For over six decades, researchers have observed the monkeys on this offshore islet in southern Japan; it is the longest continuing study of any nonhuman primate species (Kawamura 1959; Matsuzawa 2015). From 1948 to 2016, 627 individuals have been recorded in total, spanning many generations (Takahashi et al., pers. comm.). Imanishi and colleagues from Kyoto University pioneered three new methods in primatology: long-term behavioral monitoring, provisioning to habituate subjects for closer observation, and individual identification. Serendipitously, Imo, a juvenile female, invented ‘sweet potato washing’ in 1953, and wheat washing in 1956, and the spread of these behaviors throughout the group is a textbook example of spontaneous nonhuman culture, as followed from inception (e.g., Boyd and Silk 2009). This unparalleled collection of longitudinal data on these behaviors over more than 60 years allows a unique opportunity to investigate changes over many generations (Matsuzawa 2015). The potential for CC at Koshima has been asserted before, but only in brief and general terms (Avital and Jablonka 2000; Jablonka et al. 2014).

We aim to re-evaluate the claim that no CC occurs in nonhumans. We scrutinize proposed criteria for assessing CC, based on a recent review (Dean et al. 2014). Tackling CC requires an operational definition, so we devise a framework for revisiting and re-assessing the data from *Macaca fuscata* at Koshima.

### Criteria for cumulative culture

To examine the extent or absence of CC across species, Dean et al. (2014) sought to assess the behavioral and cognitive repertoires of species, mostly primates, in the wild. Their assessment entailed a two-step process. The first step was to establish a trait as cultural using the ‘method of exclusion’ (Whiten et al. 2001). This we take as given for the Koshima macaques, as one of us (McGrew 1998, 2009) has argued extensively.

Dean et al.’s second step required the trait in question to be *cumulative*: that there is direct evidence that the trait has changed over time in a directional or progressive manner, resulting in an enhanced level of *complexity*. Following Tennie et al. (2009), they stated that to be deemed cumulative, a behavioral trait must go beyond what a single individual could have invented alone (Dean et al. 2014, p 5). According to these criteria, no nonhuman species reviewed by them passed the test.

### Operationalizing cumulative culture

In order to evaluate the claim that CC is a phenomenon that is evolutionarily unique to humans (Tennie et al. 2009, p 2405), CC needs to be defined more explicitly and precisely, thus allowing systematic, quantitative, and explicit comparisons across species. CC has been defined often in recent years, but most definitions are imprecise and make no attempt to be operational (empirically testable), such as: “…innovations are progressively incorporated into a population’s stock of skills and knowledge, generating ever-more-sophisticated repertoires” (Shipton and Nielsen 2015, p 332). We more pragmatically define CC as a modification (change in the sequence or form of behavioral elements) of a cultural trait (i.e., acquired via social learning) that enhances its complexity, efficiency, security, or convenience. We acknowledge that efficiency, being a broad variable, may be too general, thus we specify two aspects of efficiency with obvious adaptive value: security and convenience. Each of these four enhancements requires more discussion. Finally, as used above, modification should be distinguished from ‘step-wise traditions,’ as proposed by Tennie et al. (2009).

### Efficiency and complexity

Dean et al. (2014) distinguished between efficiency and complexity, saying that either can be used systematically to ascertain CC, though few have tackled exactly how to measure these features. Here, we define efficiency as ‘net benefits per unit time of performance of a behavioral pattern.’ Thus, ‘net benefits’ represents the composite, proximate pay-off of energy, time, and risk accrued by an organism. For example, efficiency can be quantified as the amount of food consumed or number of feeding events per unit time, as has been done in studies of chimpanzees in captivity (Yamamoto et al. 2013; Davis et al. 2016) or in nature (McGrew and Marchant 1999; Sanz and Morgan 2009). On a wider, proximal front, efficiency can refer to greater security, convenience, comfort, even pleasure (e.g., Stewart et al. 2007), but no one has attempted to quantify this (so far as we know).

For complexity, behaviors can be understood as hierarchically organized programs of action, or ‘cultural recipes’ in which a series of dependent actions and subgoals are performed to achieve an overall end goal (Charbonneau 2015). The action chain of a hierarchically organized sequence can be broken down (parsed) into separate units, in which each unit is “a single action that results in an observable change to an item” (Byrne and Byrne 2001, p 503). Although fluid and continuous behaviors may be split or lumped arbitrarily differently in such parsing, the hierarchical and sometimes recursive unfolding of these actions can be quantified as a measure of the cognitive complexity of a task (Byrne and Byrne 2001; Byrne 2007). Such systematically structured chains appear to be transmissible between individuals (Claidière et al. 2014).

### Modification

Dean et al. (2014) distinguished accumulation from modification. They defined the former as “…addition of knowledge or behavior patterns to the behavioral repertoire of an individual or population,” (p 4) such as the addition of a new food item to a diet. For example, if a chimpanzee that already eats oranges then adds lemons (Takahata et al. 1986), this is just an accumulative augmentation of another citrus fruit to the diet. Such dietary enlargement does not constitute CC, as it does not entail the modification of a trait so that complexity or efficiency is increased. However, Dean et al. did not specify exactly what modification is. For example, if a chimpanzee cracks a nut using a wooden hammer when it previously only used stones, is this accumulation or modification? The new raw material, wood, might have superior qualities, such as being in more plentiful supply, making it more efficient to obtain. That is, the change of hammer type could represent CC, or it might be just the substitution of another raw material to produce an alternative percussor. Here we define modification as a change to the hierarchical sequence of cultural acts (i.e., behavior acquired by social learning), by addition, deletion, or substitution, which increases the effectiveness of completing a task or attaining a goal. By this behavioral standard, a change from stone to wooden hammers would be only accumulative, not CC (see Luncz et al. 2015 for reporting of stone–wood hammer choice and change).

### Japanese macaques

We now present key ethnographic data from a nonhuman primate species that is a potential case of CC. Japanese macaques inhabit a wide latitudinal range, from the subtropics to snowy mountains, making them useful for both inter- and intragroup comparisons of behavior. Japanese monkeys are behaviorally flexible and innovative, displaying stone handling (Nahallage et al. 2016), aquatic thermoregulation (Zhang et al. 2007), exploitation of marine resources (Leca et al. 2007b), specific forms of social interaction (Nakagawa et al. 2015), and food washing (Hirata et al. 2001). Of these, only for SH has the case been made for CC (Leca et al. 2012). Decades of data show cultural change, with the repertoire of SH elements increasing in number and diversity, but this seems to reflect accumulation rather than modification resulting in clear, cumulative progression. No evidence has been presented that SH is adaptive (functional), which suggests no increase in efficiency, etc. All of the SH variants are independent, simple behavioral patterns that lack complexity, so we exclude SH as exemplifying CC. Although the Koshima macaques are well documented and often cited in cultural primatology (de Waal 2001), their basic ethnography has usually been inexplicably ignored; here we seek to clarify their status.

**Fig. 1:**
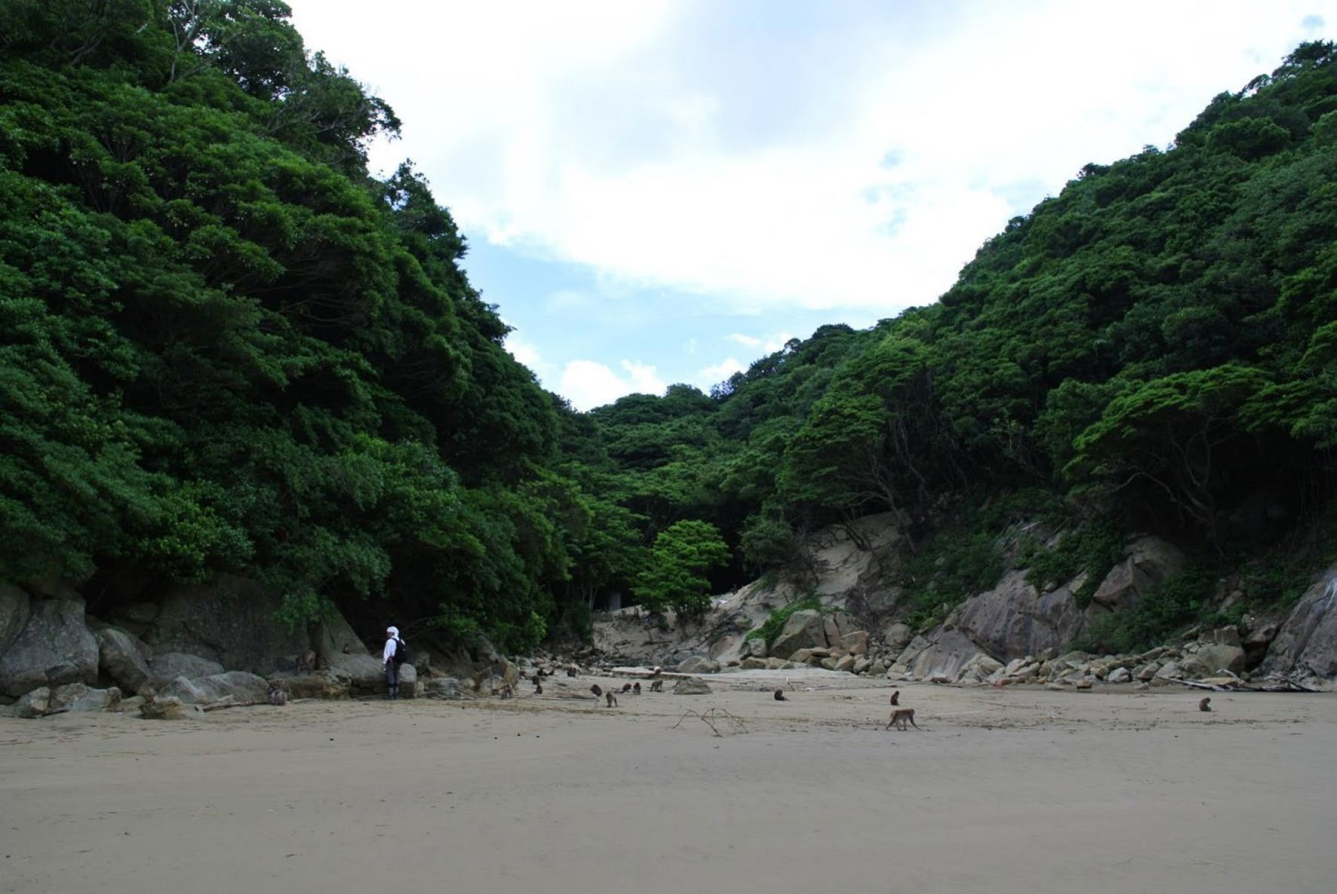
View from shoreline of the beach and forest on Koshima island (Photo by Akiko Takahashi)

**Fig. 2:**
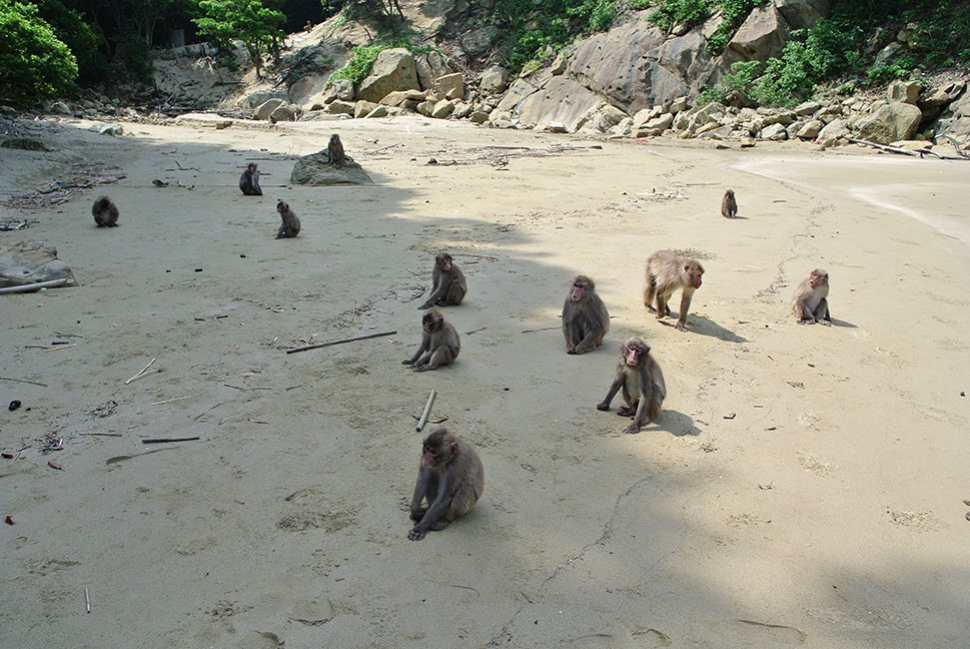
Japanese macaques on the beach at Koshima (Photo by Akiko Takahashi)

Koshima is an islet of 32 ha that is 300 m off the Kyushu mainland, in Miyazaki Prefecture, Japan (Watanabe 2001) (Fig. 1). The island has two main ecotypes: a hilly area (reaching 113 m asl) covered in thick evergreen forest, and a sandy beach and shoreline on the west side of the islet, where the macaques have been provisioned since 1952. A freshwater stream runs from the forest through the beach to the sea. The macaques have always lived in the forested areas, but after provisioning started they began to emerge intermittently to forage on the shore, where they still spend much of their time (Watanabe 1994) (Fig. 2).

### Sweet potato washing

**Fig. 3:**
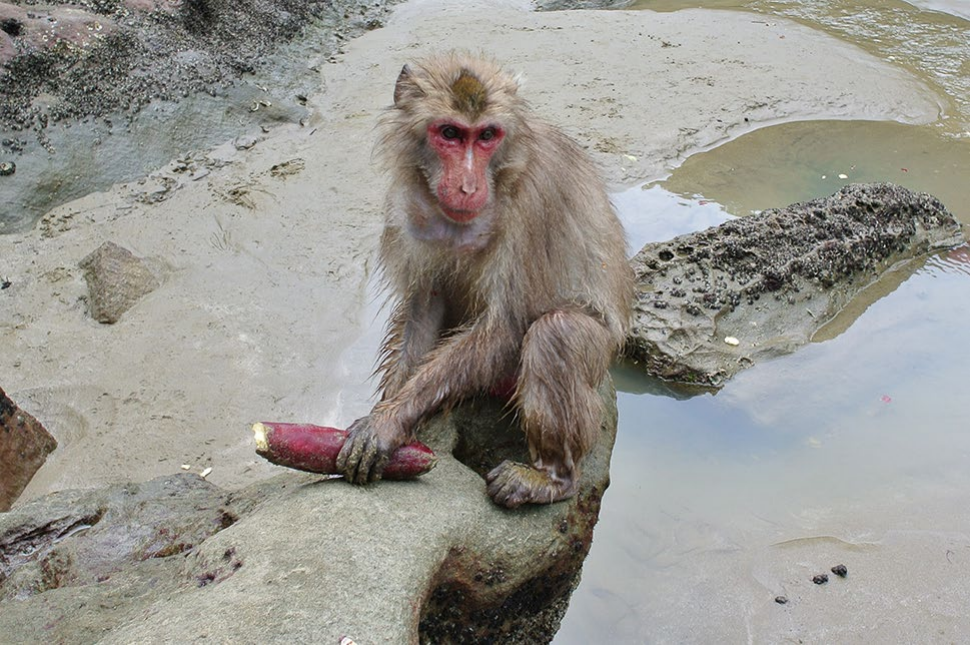
Sweet potato washing on the beach at Koshima (Photo by Akiko Takahashi)

Food washing represents hierarchically organized sequences of behavior, and has been observed elsewhere in macaques (e.g., *Macaca fascicularis*; Tan et al. 2016). At Koshima, researchers began provisioning to tempt macaques onto the beach for clearer observation conditions. The macaques were given mainly two staples: unwashed sweet potatoes were dumped and unhusked wheat grains were scattered on the sand. (Wild monkeys are well known to be crop-raiders that “grub out” sweet potatoes and pilfer rice from farmers’ fields; Kawamura 1972.) Initially, the macaques used their hands or body hair to brush away sand from the potatoes (Watanabe 1994). However, in 1953, a 1.5-year-old juvenile female named Imo started ‘sweet potato washing’ (SPW) (‘dip and brush’ in Table 1), and the behavior quickly spread to other individuals in the group (Kawai 1965). Four phases of transmission followed. SPW initially spread by horizontal transmission among some of Imo’s immature peers. Then came transmission from young to old vertically upward from child to mother, and obliquely from younger to older siblings, followed by horizontal transmission to other adults. As SPW became more established, it spread from parous females to their offspring via downward vertical transmission (Hirata et al. 2001; Huffman and Hirata 2003) (Fig. 3).

**Table 1:** Sweet potato processing chronology after provisioning at Koshima began in 1952. Kawai et al. (1992), Watanabe (1994), and Hirata et al. (2001)

Stages of apparent cultural change	Year first observed	Description of acts	Cumulative improvement
1. Brush	1952	Brush sand brush off with hand or fur	Cleaner foodstuff reduces wear on teeth from sand. Hygienic treatment may reduce risk of parasites
2. Dip and brush	1953	Dip potato in stream with one hand, and brush sand off with other	Washing more effective at removing sand, grit and soil than dry ‘brush’ variant
3. Immerse and roll	1955	Potato immersed and rolled underwater in stream	More vigorous treatment more effective at removing sand, etc. than variants 1 and 2
4. Rinse saltwater	1957	Wash potato in sea water	Wave action removes more sand, grit and soil. Flavour of potato enhanced (gustation)
5. Dip and gnaw	1958	Dip potato in sea water between repeated bites	Flavour of potato further enhanced bite-by-bite
6. Scavenge	1983	Gather pieces dropped/discarded by others	Reduced labor as less time and energy spent washing. Less risk of food being pirated?
7. Plunder	1983	Attack/threaten rinser and rob of cleaned potatoes	Reduced labor as less time and energy spent washing. Bigger portions of potatoes than 6
8. Private pool	1983	Dig own separate, more secluded pool for rinsing potato	Solitary eating decreases risk of scavengers/plunderers. Less stress means less hurried eating

Soon, SPW diversified to new variations (detailed in Table 1). It moved from fresh to salt water, from flinging to hand-held, then eventually to individual pools dug in the sand. Some changes, such as scavenging from others, seem less surprising, but there are no reports of such scavenging before provisioning began. So, we cannot know the relevant details of its emergence, but researchers at the time considered it notable enough to record as novel. These changes comprised seven progressive, cumulative steps, but not necessarily linear ones. Complexification need not be sequential, as diverse behavioral patterns may cross-fertilize one another in a kind of synergistic process (yet unstudied). For example, although scavenging and pirating by individuals may not be cumulative steps, these behaviors may have driven modifications for labor saving and increased protection of food (Kawai et al. 1992). Although SPW initially was hypothesized to enhance the palatability of the potatoes, recent findings show that SPW correlates with lowered geo-helminthic infection (Sarabian and Macintosh 2015). Emergence of new hygienic variants (e.g., from simply dipping and brushing to immersing and rolling) may represent (unintentional but potentially useful) cumulative progress in reducing the risk of acquisition of harmful parasites.

### Wheat washing

**Fig. 4:**
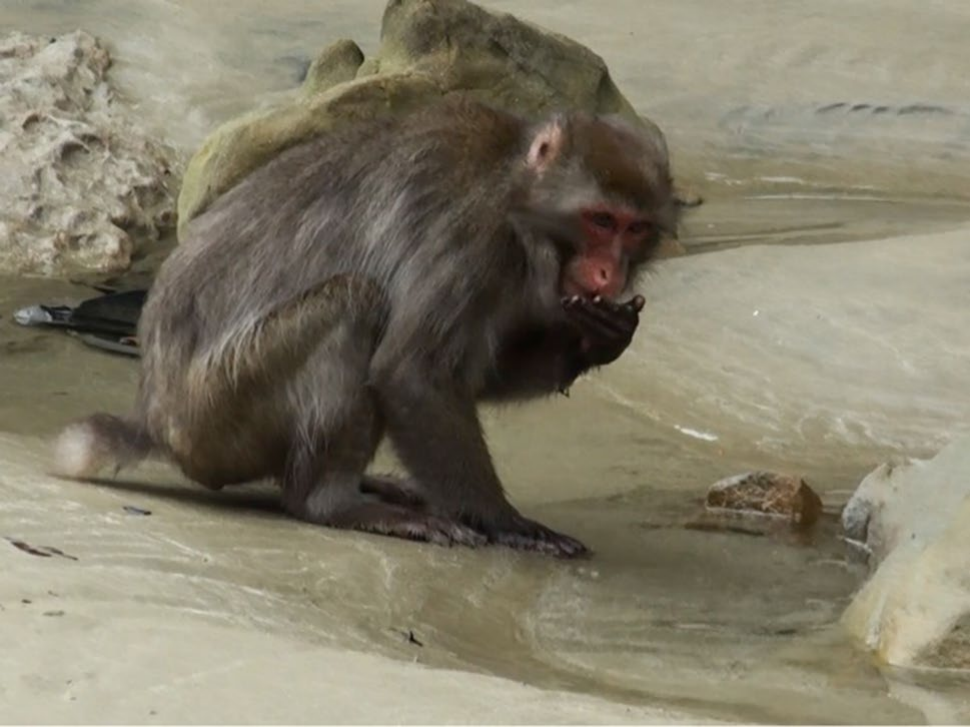
Japanese macaque washes wheat on Koshima beach (Photo by Akiko Takahashi)

‘Wheat washing’ (WW, also called ‘sluicing’ or ‘placer mining;’ Hirata et al. 2001) entails wheat grains being scattered/dropped/rinsed in water. Initially when wheat grains were scattered on the beach, the monkeys painstakingly picked up the individual grains one-by-one with thumb and forefinger opposition. The first behavioral variant emerged in 1956, when Imo picked up a mixture of wheat grains and sand from the beach, carried this mixture to the water’s edge, and flung it into the water. The sand sunk to the bottom and separated from the wheat, which floated and was scooped off the surface. This invention of WW followed a similar transmission process to SPW, with initial spread among infants through play relations, their siblings, and mothers, and—after they matured and reproduced—vertically down to their offspring (Hirata et al. 2001) (Fig. 4).

After Imo’s innovation, more enhancements were added to the repertoire of WW (Hirata et al. 2001; Kawai et al. 1992; Watanabe 1994) (Table 2 gives details of the chronology). In 1959, specialized ‘snatchers’ reduced their own labor costs by threatening and plundering the washed wheat of other individuals. Other labor-saving or security-increasing innovations included scavenging lost grains floating downstream from other wheat-washers (‘collecting’). With the accrued benefits of increased energy pay-offs from these subsistence strategies, population numbers swelled. In 1972, provisioning was reduced, leading to declines in population size. However, when the provisioning was reduced, the diversity of WW increased (Kawai et al. 1992; Watanabe 1994), and its techniques increased in complexity and efficiency. Having initially thrown the grains into the water, the macaques began to use a more secure hand-held technique, ‘dribbling,’ followed by ‘sweeping,’ ‘screening,’ and ‘mobile sweeping.’ Finally, individuals began to dig private pools in the sand for more sequestered, focused rinsing. These simple but effective measures protected against pirating snatchers and also reduced inadvertent loss of grains. Despite the end of regular provisioning in 1973, young monkeys still engage in WW (authors’ observations), as obtaining wheat 2–3 times a week is enough to sustain WW (Takahashi et al., pers. comm.).

**Table 2:** Wheat washing chronology after provisioning at Koshima began in 1952. Kawai et al. (1992), Watanabe (1994), and Hirata et al. (2001)

Stages of apparent cultural change	Year first observed	Description of act	Cumulative improvement
1. Throw	1956	Carry and drop sand/wheat mixture into water, separating wheat from sand. Skim floating wheat off surface	Grains separated from grit, so more easily consumed. Cleaner foodstuff saves wear on teeth and may reduce risk of parasites
2. Attack and plunder	1959	‘Muggers’ threaten and attack wheat washer and take grains	Labor-saving, as less energy expended collecting and washing
3. Scavenge	1962	Collect wheat grains floating downstream or in sea	Labor-saving, as less energy expended collecting and washing
4. Dribble	1970	Grasp sand and wheat mixture in hands, then repeatedly drop small amounts into water to prevent snatching by others	More secure processing against attacker/plunderers. More controlled to reduce loss of grain in water current or waves
5. Sweep	1971	Sweep wheat grains next to water’s edge by hand into water	Labor-saving: less energy expended collecting and washing
6. Screen	1974	Grasp mixture in hands, then shake in water. Sand removed with grains remaining in clenched fists	More controlled than throwing. Increased security against attacker/plunderers
7. Mobile screen	1974	Walk into water while grasping mixture in hands while screening	More controlled than throwing. Increased security against attacker/plunderers
8. Private pool	1983	Dig small depression in sand, then sweep wheat grains into resulting pool and skim off surface	Increased security against scavengers/plunderers. Labor-saving, and more controlled than the other variants—loss of wheat grains prevented

### Evaluating the Koshima findings

Some critics (e.g., Laland and Hoppitt 2003) have discounted the Koshima findings because they emerged from provisioning. Opportunities offered by artificial feeding apparently triggered Imo’s innovation, but humans made no further contribution to the monkeys’ cultural evolutionary change. De Waal (2001, p 207–209) seems to have refuted this criticism, based on first-hand reports from Koshima. Researchers did not devise an experimental study; rather, they monitored the serendipitous appearance of behavioral novelty. The monkeys currently are provisioned with no more than 3 kg of wheat overall, 3 times a week; this supplement forms only a small proportion of their overall diet, most of which is natural (Takahashi et al., pers. comm.). The monkeys range freely in nature, so provisioning is merely an affordance provided by humans that the monkeys have exploited innovatively after encountering a newly available but intermittent food resource.

Other criticisms are that the behaviors shown by the Koshima monkeys are simple patterns common to all macaques, and so are not beyond what each macaque could learn individually (Visalberghi and Fragaszy 1990; Tomasello 1999), and that behavioral innovations spread too slowly to generate cumulative change (Galef 1992, but cf. de Waal 2001, p 209–210). The initial behaviors of hand-rubbing potatoes and picking up individual wheat grains may be basic to all macaques (although we have seen no evidence presented for this claim). However, the later Koshima behaviors, entailing more efficient and complex action, seem not to have been reported for any other species of *Macaca*. Instead, the emergent, later behaviors were unexpected and seem to show innovation and enterprise. The speed of diffusion or lack of it needs to be compared with the spontaneous spread of other habits in other natural primate populations before assessing whether or not it was slow. Comparative data on diffusion rates of behaviors in Japanese macaques indicate that the behavioral type rather than purely the social context in learning best explains varying rates of diffusion (Huffman and Hirata 2003).

Finally, critics claim that SPW and WW behaviors were learned either individually by trial and error or by local/stimulus enhancement, in which one individual’s behavior at a locality ‘enhances’ the stimulus, increasing the probability that a similar discovery will be made by another individual. Essentially, the claim is that each monkey ‘reinvented the wheel’ (Tomasello 1999), and the behaviors therefore might not be culturally transmitted (dependent on one’s criteria for transmission). Thus, according to this viewpoint, the behavior of the Koshima macaques changed sporadically, even randomly, perhaps through a ‘drift-like’ process (Koerper and Stickel 1980), rather than by a progressive ‘ratchet-like’ process. At best, the critics say, macaque innovations such as SH are corruptions of existing behaviors that are “inaccurately transmitted between individuals without any further addition of complexity” (Dean et al. 2014, p 8). However, the published ethnographic data from the monkeys show patterns of spread affected by age, sex, and kinship, but not random appearance. Different matrilines showed preferences for specific variants of SPW and WW (Hirata et al. 2001). Not surprisingly, the snatching of others’ grains in WW was performed more by monkeys of dominant lineages (Kawai et al. 1992). Regardless of the social learning mechanisms involved (see below), enough change has occurred to suggest potentially cumulative increases in complexity and efficiency of the washing behaviors, as derived from 60 years of data from Koshima.

## Discussion

The descriptive post hoc data presented here can be only suggestive, not conclusive. Qualitative reports need to be succeeded by quantitative testing of hypotheses. This is clear in principle but uncertain in practice, being dependent on current (not historic) conditions at Koshima. But, in the meantime, the qualitative reports reflect topical issues relating to CC, as outlined below.

### High-fidelity transmission

The predominant social learning mechanisms of Japanese macaques remain unclear, and little research has been done on captive populations (Hirata et al. 2001). Nahallage et al. (2016) suggest that SH is transmitted through stimulus enhancement and response facilitation. However, even if food-washing behaviors spread through simple forms of social learning such as these, they seem to have enabled the occurrence of CC. Further, recent evidence from studies of other nonhuman species indicates that culture occurs with simpler transmission mechanisms than previously thought (Logan et al. 2016). This suggests that high-fidelity learning, which is so often stated to be essential to CC (Lewis and Laland 2012; Tomasello 2016) may not be necessary (Sasaki and Biro 2017). Recent experimental evidence on humans suggests that high-fidelity social learning is useful but not necessary to generate CC (Caldwell 2015; Zwirner and Thornton 2015; but cf. Wasielewski 2014). Observational data from the Aka hunter-gatherers of the Congo basin suggests that most skills are learned by passive observation, not necessarily by direct teaching or imitation (Hewlett et al. 2011).

### Individual and social learning

Although the analysis of Dean et al. (2014) was acute, their criteria for innovation seem overly restrictive. In particular, their criterion that a trait must be beyond individual innovative capacity is problematic, as hypotheses that are framed in the negative, i.e., ‘X cannot do something’ cannot be verified empirically, as it is logically impossible to prove the absence of something. It also seems nonsensical, as each actual innovation expands the imaginable limits of what any individual or species can or cannot do, ad infinitum. Nonhumans repeatedly surprise us by their inventiveness, making it impossible to say a priori what could or could not be achieved (Kummer and Goodall 1985; Nishida et al. 2009). But what of space shuttles, or mobile phone technology? The argument is that these cases would be impossible to invent de novo, and nonhumans have no such innovations. Although these cases from modern industrial society are impressive, such arguments commit the ‘Space Shuttle Fallacy’ (McGrew 2004). Most individual humans have not done these things, and using this criterion would exclude populations of *Homo sapiens* (e.g., traditional hunter-gatherer societies). Citing a hypothetical “zone of latent solutions” in nature, demarcated by the upper boundary of a species’ cognitive skills (Tennie et al. 2009), does not help matters unless its validity is empirically testable in situ. This is a formidable challenge, and we await operational criteria (rather than proposed features) that will be applicable in an ecologically valid context.

### Culture evolves and devolves

Contrary to the ratchet effect, proposed by Tennie et al. (2009), culture does not seem to evolve in the simple, unidirectional progression that the analogy implies, and in theory there is no reason why CC should always be heading for more complexity or efficiency. Culture change does not equal CC; environments or demographics change and culture responds (Kolodny et al. 2015). For example, over millennia during the Holocene, Tasmanian hunter-gatherers lost valuable skills and technologies, apparently due to the population bottleneck from rising ocean levels at the end of the last glacial epoch (Henrich 2004). Critics claim that nonhuman traditions are few, predictable, and transient, but just as in human culture—under suitable conditions—nonhuman cultural traits in nature apparently increase in prevalence, complexity, and efficiency, as evidenced by the Koshima monkeys. Their behavior was not linearly progressive, as the ratchet implies, but often flexible, interchangeable, and intermittent (Avital and Jablonka 2000; Jablonka et al. 2014). Those ‘stages’ did not unfold one after the other; variants multiplied over time. Cultural variants seem to develop and fluctuate based on various factors, such as the inextricable interactions of environment, demography, and perhaps even gene frequencies. The Koshima monkeys show that with minimal exposure to wheat and sweet potatoes, cultural traits emerge and persist, apparently with improvement. However, as we have stressed above, hypothetical assertions require empirical testing to be conclusive.

### Time depth

If nonhumans generate CC, why is there so little evidence of it? The Koshima data suggest that long-term data may be required to detect CC. Most studies and comparative data from the wild are from relatively brief snapshots (months or years rather than decades), which may be why nonhuman CC is so elusive. Time per se is not the issue, but rather the long generation times of large-brained, K-selected mammals, which may mean that decades are needed to compile the data. Many generations have followed on at Koshima after Imo’s innovations, but tracing the precise lineages for each behavioral pattern across multiple generations remains to be done. We know of a few such efforts to use chronological, archival data retrospectively to reconstruct cultural change (e.g., diffusion of ant fishing in Gombe chimpanzees, O’Malley et al. 2012), but we know of no such efforts to infer past CC.

Whether or not CC is unique to humans also depends greatly on how the phenomenon is defined. As with traditional arguments used against culture in nonhumans, using social learning processes (e.g., teaching and imitation) both to define and as evidence for CC is logically flawed, and restricts CC to humans; for this position to be valid, high fidelity mechanisms must be shown to be necessary for CC, not simply more effective (Gruber 2016). Definitions vary, but so long as they are precise, explicit, and operational, the question of CC in nonhumans can be addressed in a comparative framework. Although cultural evolution is more rapid than genetic evolution, a challenge for comparative studies of CC is that macroevolutionary change in human evolution seems to operate at several orders of magnitude faster than in other taxa. Despite this challenge, the macaque evidence seems to show that 60+ years of study could be enough to trace progressive change. Imo’s initial innovations enhanced the performance of basic subsistence activities and triggered the transmission and modification of other variants, which seem to have increased in complexity, security, convenience, and efficiency.

## Conclusion

So, why have the Koshima data, which have long resided in the public domain, been ignored in current discussions of CC? Perhaps present-day commentators have not carefully read the original, older ethnography (Kawamura 1959; Kawai 1965) or detailed synthetic accounts of its progression (Itani and Nishimura 1973), even if they sometimes cite these primary or secondary sources. Or, they cite later publications (Hirata et al. 2001; Kawai et al. 1992; Watanabe 1994) without going back to the original reports. Language is not a barrier to access: we have used only English-language sources, but we did not find any additional information available in Japanese-language publications or data archives.

Our review of Koshima ethnography indicates that the food-washing behaviors of the monkeys may have accumulated in complexity and efficiency, consistent with definitions of CC. This suggests that the evolutionary roots of CC are deep in the primate clade. Our reprise of the overlooked Koshima ethnographic record is not conclusive, as the precise changes in complexity and efficiency of the different washing behaviors remain to be tested. An example of how these ideas might be tested would be to survey which variants of the washing behaviors still exist today, and if so, determine which of the enhancements are more frequent when there are more monkeys on the beach or in close proximity. However, these findings at least appear to call into question the overwhelming current received wisdom that nonhuman animals cannot build upon behavioral improvements made by previous group members, and challenges the idea that only humans have history and cultural evolution that other animals lack (Mesoudi 2011). We believe that these data may dispel the idea that human uniqueness is the best null hypothesis and should encourage open-ended future research on nonhuman CC, at least for investigators seeking its evolutionary roots in ourselves. We hope to see human ethological studies of CC in operation in the real-world, spontaneous behavior of *Homo sapiens*.
